# The catechol-o-methyltransferase Val^158^Met polymorphism modulates the intrinsic functional network centrality of the parahippocampal cortex in healthy subjects

**DOI:** 10.1038/srep10105

**Published:** 2015-06-09

**Authors:** Xiaolong Zhang, Jin Li, Wen Qin, Chunshui Yu, Bing Liu, Tianzi Jiang

**Affiliations:** 1 Brainnetome Center; 2 National Laboratory of Pattern Recognition, Institute of Automation, Chinese Academy of Sciences, Beijing 100190, China; 3 Department of Radiology, Tianjin Medical University General Hospital, Tianjin 300052, China; 4 Queensland Brain Institute, The University of Queensland, Brisbane, QLD 4072, Australia; 5 Key Laboratory for NeuroInformation of Ministry of Education, School of Life Science and Technology, University of Electronic Science and Technology of China, Chengdu 610054, China

## Abstract

The influence of catechol-o-methyltransferase (COMT) Val^158^Met on brain activation and functional connectivity has been widely reported. However, voxel-wise effects of this genotype on resting-state brain networks remain unclear. Here, we used resting-state fMRI and eigenvector centrality to examine the effects of COMT Val^158^Met genotypes on the connection patterns of the brain network and working memory (WM) in healthy, young Val/Val and Met carrier subjects. There were significant differences in the performance level on the 2-back WM task between the different COMT genotypes: Val/Val individuals exhibited a higher correct rate compared to the Met carriers. A two-sample t test was used to examine the differences in the eigenvector centrality maps, using age and gender as covariates of no interest, between the Val/Val and Met carriers. We found that the Val/Val individuals exhibited significantly higher eigenvector centrality compared to the Met carriers in the left parahippocampal cortex. Furthermore, a significantly positive correlation between the mean eigenvector centrality of the significant cluster and the correct rate of the 2-back WM task was observed. By using a voxel-wise data-driven method, our findings may provide plausible implications regarding individual differences in the genetic contribution of COMT Val158Met to the brain network and cognition.

The catechol-O-methyltransferase (COMT) gene, which contains a well-studied functional polymorphism (Val^158^Met), plays a critical role in central dopamine function through the degradation of the COMT enzyme^1^. Behavioral findings suggest that the Val^158^Met polymorphism is related to a number of executive functions and memory performance, including n-back working memory (WM) tasks^2^,^3^. Several studies have reported that Val homozygous subjects show greater activation in the prefrontal cortex (PFC) for the same level of performance, which indicates less ‘efficiency’ compared to other COMT genotypes^4^,^5^. Moreover, the impact of COMT on brain activation during cognitive tasks is not limited to the PFC; the impact extends into the cingulate cortex, hippocampus, ventral striatum and amygdala^6^,^7^. COMT also modulates the functional connectivity in different brain regions during task performance, and its precise effect varies with the task performed and the neural circuitry involved^6^,^8^,^9^,^10^. Besides these task-based studies, previous studies have also examined the effect of COMT on the default mode network at rest^11^,^12^. The investigation of COMT Val^158^Met effects from the perspective of brain networks may provide a systematic understanding of the genetic effects.

Although the majority of studies have focused on task-related brain activation, resting (‘spontaneous’) neuronal activity in the absence of external stimuli is attracting wide attention^13^. Resting-state functional connectivity, which is measured by resting-state functional magnetic resonance imaging (fMRI), examines temporal correlations of spontaneous neuronal activity across brain regions and has been proven useful for mapping brain networks^14^,^15^ and predicting individual cognitive performance^16^,^17^. Common techniques to study resting-state functional connectivity include seed-based correlation^18^ and independent component analysis (ICA)^19^,^20^, which require prior knowledge to identify seed regions (seed-based analysis), specify the number of independent components and make assumptions regarding what constitutes a valid network (ICA)^21^. To avoid any selection bias, we propose to apply a particular type of graph-based method, eigenvector centrality (EC), to all voxels of the entire brain^22^. The EC weights nodes according to their number of connections as well as the quality of their connections within the network. Google’s “PageRank” algorithm, is effective as part of a search engine, is a implementation of EC^23^. We supposed that EC could be effective in analyzing network properties of the human brain. Therefore, we aimed to use the EC measure to investigate the voxel-wise effects of COMT Val^158^Met on resting-state brain networks.

In this study, we used a voxel-wise data-driven method to examine the influence of COMT Val^158^Met on the intrinsic brain networks in a large sample of young healthy subjects (N = 287). The EC of each voxel throughout the whole brain was obtained for each individual based on resting-state fMRI data. The differences in the EC maps between the different COMT genotypes were subsequently examined. The relationships between COMT Val^158^Met, network centrality and working memory performance were further analyzed to provide a potential schema of the genetic variant, brain networks, and cognitive function.

## Results

### Demographic and Genetic Characteristics

Two hundred eighty-seven young healthy subjects were included in the current study. We merged the Met homozygotes and heterozygotes into one group of Met carriers because the frequency of Met homozygotes was 5-6 times lower than that of the Val homozygotes. There was no significant main effect of genotype on any of the demographic variables, such as gender, age or education level. However, a significant (P = 0.019) main effect of the COMT genotype on the correct rate of the 2-back WM tasks was identified, but an effect of the COMT genotype on 3-back WM performance (P = 0.588) was not found. Detailed demographic and cognitive data are summarized in Table 1. We also summarized the detailed demographic and cognitive data of the Val homozygote, the Met homozygote and the heterozygote groups (Supplementary Table).

### Eigenvector Centrality Map Differences

**Figure 1:**
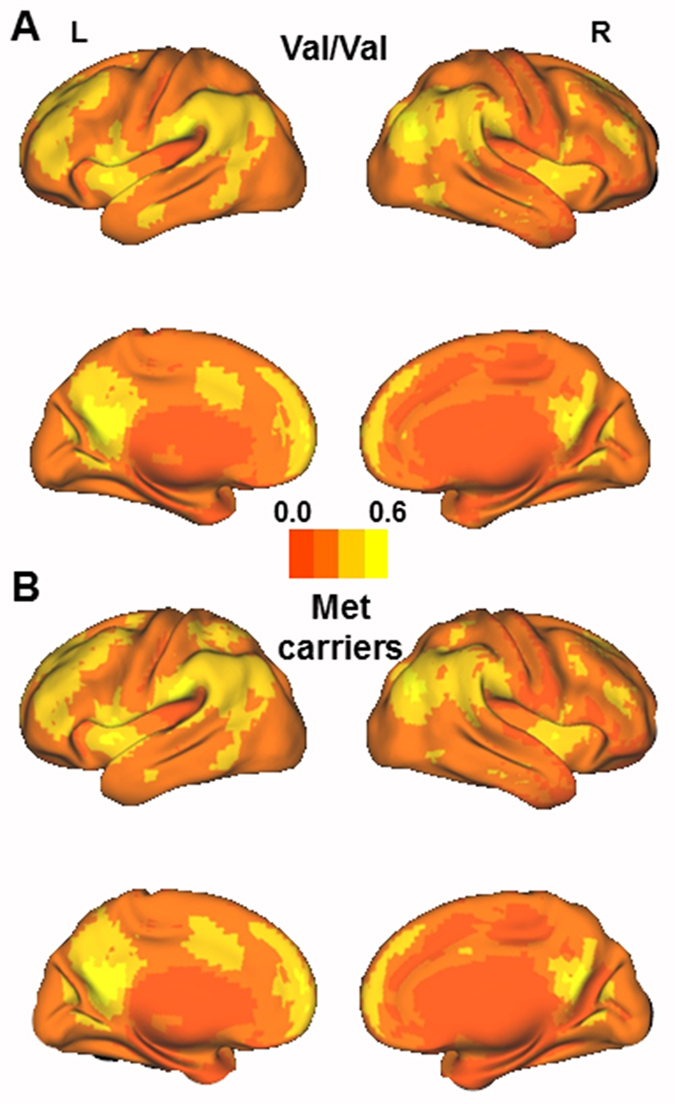
The mean eigenvector centrality maps for Val/Val individuals (**A**) and Met carriers (**B**).

**Figure 2:**
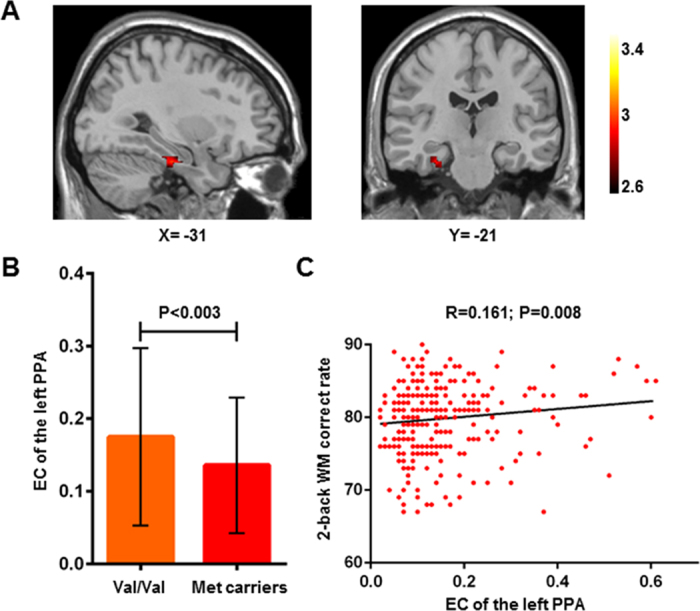
Effects of COMT Val^158^Met genotypes on the eigenvector centrality of the brain network and working memory performance. (**A**) A two-sample t test of eigenvector centrality maps revealed significantly (P < 0.05, corrected) higher centrality in the left parahippocampal cortex (peak voxel MNI coordinates: X = −24, Y = −20, Z = −28; T = 2.939; cluster size = 27) in the Val/Val individuals than the Met carriers. (**B**) For illustrative purposes, the mean eigenvector centrality within the significant cluster is displayed in the bar graph for the two different genotype groups (mean ± SD). (**C**) Individual eigenvector centrality within the significant cluster is significantly (P = 0.008) positively correlated with the individual 2-back WM correct rate. The P value is shown with age and gender as covariates in the association analysis. PPA, parahippocampal cortex; WM, working memory.

The mean EC maps of different COMT genotypes are shown in Fig. 1; the patterns of EC maps of the whole brain were similar between the different genotypes. We used two-sample t-tests to examine the differences in the EC values between the two genotype groups; we determined that the subjects who were Val homozygotes exhibited significantly higher EC in the left parahippocampal cortex (peak voxel MNI coordinates: X = −24, Y = −20, Z = −28; T = 2.939; cluster size = 27) compared to Met carriers (Fig. 2A,B).

### ROI Analysis

We extracted the mean EC of the cluster (27 voxels) with significant differences in the left parahippocampal cortex (Fig. 2B). We subsequently found a significantly positive correlation between the extracted mean EC and the correct rate of the 2-back working memory tasks when age and gender were included as covariates (P = 0.008, R = 0.161, Fig. 2C).

## Discussion

Based on resting-state fMRI data and EC maps, we found that COMT Val homozygous individuals exhibited significantly higher EC in the left parahippocampal cortex than Met carriers. Moreover, we demonstrated that the EC of the left parahippocampal cortex was significantly correlated with the correct rate of the 2-back WM task. These findings suggest that COMT Val^158^Met affects the intrinsic functional connectivity between the left parahippocampus and other brain regions. By using a voxel-wise data-driven method, our findings may provide plausible implications regarding individual differences in the genetic contribution of COMT Val^158^Met to the brain network and cognition.

In our study, a verbal WM task was employed, and a significant difference in 2-back WM performance between different COMT genotypes was observed. However, the significant difference was not found in the 3-back WM task, which might have occurred because the 3-back WM task induced dopamine increase leads to the transgression of the optimum of the u-curve for Val homozygotes, thus diminishing the genotype specific differences^24^. Previous studies have shown inconsistent results regarding the relationship between COMT and N-back working memory performance, some studies have reported that one or two copies of the Met allele were associated with superior performance^25^,^26^, while other studies have failed to replicate this finding^27^,^28^. The effect of COMT on N-Back performance requires further study.

Because EC weights nodes based on the degree of their connections within the network by counting not only the number but also the quality of the connections, it takes into account the entire pattern of the network^29^ and is sensitive to different layers in the network hierarchy^30^. EC has also been shown to be robust against global physiological effects and is easily computable from the voxel-wise connectivity matrix^21^ when used as a measure of functional connectivity in resting-state fMRI. The increased EC of the left parahippocampal cortex in the Val homozygous subjects compared to the Met carriers could result from the influence of the COMT Val^158^Met polymorphism on the connections between the parahippocampus and other regions. The COMT Val^158^Met polymorphism may modulate several connections of the left parahippocampus to high-level regions, such as the hippocampus^31^, which plays an important role in memory function, or a large number of connections to medium-level areas, such as the lateral parietal cortex^32^, or to both types of areas. A previous study also underscored the importance of the parahippocampal gyrus as a mediator of the effect of COMT on resting-state functional connectivity between the anterior medial PFC and the ventrolateral as well as the dorsolateral PFC and the parahippocampal gyrus^33^. Here, we applied EC analysis to all voxels in the grey matter, which avoided selection bias^34^. While COMT Val^158^Met affects hippocampal-PFC coupling^35^,^36^, it is noteworthy that the parahippocampal gyrus constitutes the primary hub of the default mode network in the medial temporal lobe (MTL)^37^ and also represents an important input region for the hippocampus^38^, thus underlining the plausibility of our finding that differences in left parahippocampal functional connectivity between Val homozygotes and Met carriers exist even at rest.

We found that the mean EC of the cluster that exhibited significant differences between the Val homozygotes and Met carriers in the left parahippocampal cortex had a significantly positive correlation with the correct rate of the 2-back WM test. This finding may be supported by previous study that has found significant left hemispheric lateralization for verbal working memory^39^. Previous studies have also shown that resting-state functional connectivity may predict individual cognitive performance^17^,^40^. Several studies have reported that the COMT Val variant is associated with inefficient prefrontal-related functional connectivities at rest and poor cognitive performance^8^,^11^,^12^. In addition, a previous study demonstrated that Val homozygotes exhibited significantly decreased MTL and increased PFC activity during both successful relational encoding and retrieval and a disrupted connectivity between these areas compared to Met carriers^41^. Given the fMRI and lesion studies that suggest that parahippocampal regions mediate working memory for novel stimuli^42^, we suppose that the COMT Val^158^Met polymorphism affects WM by modulating the parahippocampal intrinsic functional connectivity.

Our findings demonstrated the effect of the functional polymorphism COMT Val^158^Met on the functional connectivity between the left parahippocampus and other regions at rest. However, previous studies have reported at least three COMT functional polymorphisms (rs737865, rs4680, rs165599) that impact its biological activity^43^ and certain commonly found haplotypes, which may regulate the COMT gene more strongly than Val^158^Met^44^. Thus, the effect of other functional polymorphisms and their common haplotypes requires further study. Moreover, in this study there was a trend for education years to be associated with COMT genotypes, we should match different groups for demographic data in future studies. In the present study, we used a Monte Carlo simulation for multiple comparison correction. The lack of significant intergroup differences after the FWE correction, suggests that these findings should be independently validated in future studies.

In conclusion, our findings based on voxel-wise brain network analyses demonstrated that the COMT Val^158^Met polymorphism is significantly associated with the network centrality of the left parahippocampus and individual WM performance. These findings show disrupted EC of the left parahippocampus, which indicates that aberrant connectivity between the left parahippocampus and other regions may contribute to the individual WM performance differences. However, additional biological evidence and independent validation are needed in future studies to further support the current findings.

## Methods

### Participants

We recruited 323 healthy, young, right-handed Han Chinese individuals (157 males and 166 females, mean age = 22.7 ± 2.5 years, range = 18–31 years) in this study. After a complete study description, all participants provided written informed consent. This protocol was approved by the Ethics Committee of Tianjin Medical University. All experiments were performed in accordance with approved guidelines and regulations. We carefully screened the participants to ensure that they had no history of psychiatric or neurological illness, alcohol or drug abuse, or psychiatric treatment, as well as no contraindications for magnetic resonance image (MRI) examinations. All subjects were examined using the Chinese Revised Wechsler Adult Intelligence Scale (WAIS-RC). Following the exclusion of 6 subjects without COMT Val^158^Met genotype data and 30 subjects who lacked sufficient fMRI data, 287 subjects were included in our study.

### COMT VAL^158^Met Genotyping

Genomic DNA was extracted from whole blood using the EzgeneTM Blood gDNA Miniprep Kit (Biomiga Inc., San Diego, CA, USA). For each subject, we genotyped COMT Val^158^Met (rs4680) using the PCR and ligation detection reaction (LDR) method as described in our previous study^11^.

The COMT rs4680 genotype distribution in the sample was in Hardy–Weinberg equilibrium (P > 0.05). The frequencies of the COMT genotypes are presented in Table 1. No genotype distribution differences were found between the males and females. The subjects who were homozygous and heterozygous for the A-allele (Met) were merged into a group of A-allele carriers and compared with the homozygotes for the G-allele (Val); this method has previously been used to address skewed genotypic distributions.

### Image Data Acquisition

All subjects were scanned using a Signa HDx 3.0 Tesla MR scanner (GE Healthcare; Milwaukee, WI, USA). Resting-state functional imaging was performed using a single-shot gradient-echo echo-planar-imaging (SS-GRE-EPI) sequence, which is sensitive to blood oxygenation level-dependent (BOLD) contrast, with the following parameters: TR = 2,000 ms, TE = 30 ms, field of view (FOV) = 240 × 240 mm^2^, matrix = 64 × 64, flip angle = 90°, voxel size = 3.75 × 3.75 × 4.0 mm^3^, 40 slices, and 180 volumes. During the resting-state fMRI scans, all subjects were instructed to move as little as possible, keep their eyes closed, and refrain from sleeping.

### Data Preprocessing

We checked each functional MRI from all subjects slice by slice to exclude the images with obvious inter-slice motion artifacts. Further data preprocessing was completed using the Data Processing Assistant for Resting-State fMRI (DPARSF)^45^. The following preprocessing steps were performed: 1) the first 10 volumes were discarded; 2) slice timing correction; 3) realignment of the volumes to the first volume to correct for head motion; 4) spatial normalization to a standard EPI template and resampling to 4 mm^3^ voxels; 5) spatial smoothing using an 8 mm Gaussian kernel; 6) linear regression to remove the effects of whole brain signals and linear trends; 7) temporal band-pass filtration (0.01-0.08 Hz); and 8) the multiple regression method was used to remove the potential effects of confounding factors. Subjects who exhibited a maximum displacement greater than 2 mm in any of the cardinal directions (x, y, z) or a maximum spin (x, y, z) greater than 2° were excluded from the following analyses.

### Working Memory Performance

The working memory task was performed on a computer in a quiet room outside the MRI scanner. Working memory was assessed by letter-based 2-back and 3-back tasks as previously described^46^. Briefly, the subjects viewed a series of letters that were presented sequentially; they were asked to judge whether the letter on the screen was the same as that presented two letters earlier (in the 2-back task) or that presented three letters earlier (in the 3-back task). Each task consisted of three blocks (30 trials per block). Prior to the experiment, the subjects were verbally instructed and completed a practice block to ensure that they understood the task prior to the completion of the formal tests. The number of correct trials divided by 90, namely the correct rate, was used as the index of working memory performance in the current study.

### Network Eigenvector Centrality Analysis

For each subject, we calculated voxel-wise EC maps as previously described^29^. For each individual, an N
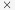
N correlation matrix (R) was computed based on the time series of each voxel throughout the whole brain; N represented the number of voxels of grey matter, and the square value of R was calculated. Here, we chose S = 1.2, which is represented by: 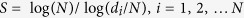; where d_i_ represents the degree of the voxel i, which was used to define the network threshold^47^. Then, an eigenvector that belonged to the normalized largest eigenvalue of the resulting weighted adjacency matrix (A) was calculated, and its entries (x_i_) provided a centrality measure for each voxel i. To ensure that the EC obeyed a Gaussian normal distribution, a Fisher’s z transformation was used for the subsequent statistical tests.

### Statistical Analysis

We used a Chi-square test to examine the gender differences and one-way ANOVA to identify differences in age, education level, and correct rate of the n-back working memory tasks between the two COMT Val^158^Met genotype groups using Statistical Package for the Social Sciences version 18.0 (SPSS, Chicago, IL, USA) for Windows. Voxel-wise two-sample t-tests implemented in SPM8 were subsequently used to map the group differences in the EC maps between the COMT Val^158^Met Val homozygotes and the Met carriers; age and gender were included as covariates of no interest. The results were subjected to a Monte Carlo simulation (n = 1000 iterations) and corrected for multiple comparisons with a significance level of p < 0.05 and a cluster size of at least 17 voxels (voxel-level threshold of P < 0.005, FWHM = 8 mm, cluster connection radius r = 5 mm; with a grey matter mask and a resolution of 4 mm^3^). This calculation was implemented using the AlphaSim procedure performed in the REST toolbox ( http://restfmri.net/forum/index.php). Finally, a two-tailed Pearson correlation was used to test the association between the EC of the resulting clusters with significant differences in EC and WM performance with age and gender as covariates. An alpha of p < 0.05 was considered significant.

## Additional Information

**How to cite this article**: Zhang, X. *et al.* The catechol-o-methyltransferase Val^158^Met polymorphism modulates the intrinsic functional network centrality of the parahippocampal cortex in healthy subjects. *Sci. Rep.* **5**, 10105; doi: 10.1038/srep10105 (2015).

## Supplementary Material

Supplementary Information Supplementary Tables S1

## Figures and Tables

**Table 1:** Demographic information of all subjects included in this study.

	**Val/Val**	**Met carriers**	**P-value**
N	137	150	
Male : Female	59:78	73:77	0.904
Age (years)	22.9 ± 2.4	22.7 ± 2.5	0.524
Age range (years)	18–29	18–29	
Education (years)	15.9 ± 2.3	15.4 ± 2.8	0.067
CorrectRate_2back	89.5 ± 5.4	88.0 ± 5.4	0.019
CorrectRate_3back	82.1 ± 6.4	81.6 ± 6.3	0.588

Values denote mean ± standard deviation or number of subjects; CorrectRate denotes the percentage correct.
