# Supplementary material for: The catechol-o-methyltransferase Val158Met polymorphism modulates the intrinsic functional network centrality of the parahippocampal cortex in healthy subjects
Source: Sci Rep. 2015 Jun 9;5:10105. doi: 10.1038/srep10105 (PMC4460568; doi:10.1038/srep10105)
Supplement: Supplementary Information — Supplementary Tables S1 [file srep10105-s1.doc]

**The** **catechol-o-methyltransferase Val^158^Met polymorphism modulates the intrinsic functional network centrality of the parahippocampal cortex in healthy subjects**

Xiaolong Zhang^1^, Jin Li^1,2^, Wen Qin^3^, Chunshui Yu^3^, Bing Liu^1,2, *^,Tianzi Jiang^1, 2,4, 5^.

**Supplementary Table 1**.Demographic information of all subjects according to COMT genotypes included in this study.

|  | Val/Val | Val/Met | Met/Met | P-value |
| --- | --- | --- | --- | --- |
| N | 137 | 123 | 27 |  |
| Male : Female | 59:78 | 57:66 | 16:11 | 0.302 |
| Age(years) | 22.9±2.4 | 22.8±2.5 | 22.2±2.5 | 0.449 |
| Age range(years) | 18-29 | 18-29 | 19-27 |  |
| Education(years) | 15.9±2.3 | 15.4±2.9 | 15±2.2 | 0.147 |
| CorrectRate_2back | 89.5±5.4 | 88.1±5.5 | 87.7±5.2 | 0.064 |
| CorrectRate_3back | 82.1±6.4 | 82.1±6.2 | 79.8±6.6 | 0.257 |

Values denote mean ± standard deviation or number of subjects; CorrectRate denotes percentage correct.
